# Integrative medicine: a tale of two clinics

**DOI:** 10.1186/1472-6882-8-32

**Published:** 2008-06-18

**Authors:** Heather S Boon, Natasha Kachan

**Affiliations:** 1 Leslie Dan Faculty of Pharmacy, University of Toronto, Toronto, Canada

## Abstract

**Background:**

Integrative medicine (blending the best of complementary and alternative medicine (CAM) with conventional medicine) is becoming increasingly popular.

**Objectives:**

The objectives of this paper are to compare and contrast the development of two teams that set out to establish integrative medical clinics, highlighting key issues found to be common to both settings, and to identify factors that appear to be necessary for integration to occur.

**Methods:**

At St Michael's Hospital (an inner-city teaching hospital in Toronto, Canada), a total of 42 interviews were conducted between February 2004 and August 2006 wi18 key participants (4 administrators, 2 chiropractors, 2 physiotherapists and 10 family physicians). At the CARE (Complementary and Alternative Research and Education) Program at Stollery Children's Hospital, Edmonton, Canada, 44 interviews were conducted with 24 people on four occasions: June 2004, March 2005, November 2006, and June 2007. Basic content analysis was used to identify the key themes from the transcribed interviews.

**Results:**

Despite the contextual differences between the two programs, a striking number of similar themes emerged from the data. The five most important shared themes were: 1) the necessity of "champions" and institutional facilitators to conceive of, advocate for, and bring the programs to fruition; 2) the credibility of these champions and facilitators (and the credibility of the program being established) was key to the acceptance and growth of the program in each setting; 3) the ability to find the "right" practitioners and staff to establish the integrative team was crucial to each program's ultimate success; 4) the importance of trust (both the trustworthiness of the developing program as well as the trust that developed between the practitioners in the integrative team); and 5) the challenge of finding physical space to house the programs.

**Conclusion:**

The programs were ultimately successful because of the credibility of the champions, institutional facilitators and the staff members. Selection of excellent clinicians who were able to work well as a team facilitated the establishment of trust both within the team itself as well as between the team and the host institution.

## Background

"It was the best of times, it was the worst of times...."[1] Charles Dickens had never heard of integrative medicine when he wrote the opening line to his classic text, but it fittingly describes the context in which integrative medicine clinics find themselves today. [2,3] There is increasing evidence that both patients and clinicians see integrative medicine as the best way to provide optimal health care. [2,4] Yet, a definition of what constitutes integrative medicine remains elusive. [5,6] which makes it difficult to determine who is (and who isn't) practicing it. Integration can occur at a variety of different levels: from patients who combine various therapies to practitioners who practice different modalities, clinics that offer a range of therapies, and health care systems that facilitate the use of multiple treatment options. [7] Dalen describes integrative medicine as both high-tech and high-touch medicine and argues that dual trained physicians are ideally placed to make this happen. [2] Others focus on the interdisciplinary nature of integrative medicine. [5,8] For the purposes of this paper, integrative medicine is defined as the interdisciplinary blending of conventional medicine and CAM with the purpose of enhancing patients' health.

Many authors have attempted to identify factors related to successful integration of CAM and conventional medicine. For example, Hsiao et al.'s interviews with a wide range of practitioners show that successful integration is related to provider attitudes toward integrative medicine, knowledge of integrative medicine, readiness to refer to other practitioners and ability of clinicians to practice more than a single modality. [6] This work highlights the multi-dimensional nature of the concept of integrative medicine, provides a conceptual model that links provider characteristics with provider behaviours and shows how they appear to be mediated by provider attitudes and knowledge [6].

There are also a number of critiques that the type of medicine being practiced by those claiming to provide "integrative medicine" are not in actuality integrated according to many definitions of the concept. [5,9] For example, Hollenberg describes the exclusionary and demarcationary closure strategies of conventional medicine practitioners in two integrative medicine clinics. Hollenberg argues that even while striving to collaborate with their CAM colleagues, conventional medicine practitioners in these clinics continue to perpetuate patterns of conventional medicine dominance by maintaining control of overall patient care and using conventional medicine language as the primary form of communication throughout the clinics. Hollenberg also describes how the CAM practitioners in the clinics employ usurpationary closure strategies by evoking their own specialized forms of knowledge and referring among themselves to increase patient flow among the CAM practitioners. [9] Boon et al. argue that most definitions of integrative medicine describe an ideal goal as opposed to a functioning program [5].

There is an increasing number of accounts of integrative medicine program developments in the literature. (i.e. [10-14]) These generally provide guidance for others planning similar projects. A brief review of these reports identifies similar success factors including: open-minded attitudes of administrators at host institutions, indicating an open-minded institutional culture. [10,13]; highly competent CAM and conventional medicine practitioners [10,11]; effective communication among team members [9-13]; sustainable environment (both physical and economic)[10,12,13,15]; and ability to fit unique needs of constituents. [10,13]. The objective of this paper is to compare and contrast the development of two teams that set out to establish integrative medical clinics. Despite the clinics' obvious differences (in one setting chiropractors were integrated into the family medicine outpatient service of a large inner city teaching hospital; in the other, a pediatric CAM consultation service was established in a large pediatric hospital along with CAM research and education programs) there were many success factors that were similar across both programs. Thus, in this paper we not only seek to confirm the findings of earlier studies, but also to begin the process of identifying factors that are necessary for integration to occur. This paper will highlight key issues found to be common to both integrative medicine initiatives, and likely common to most integrative programs.

## Methods

In this paper we compare the findings from two different applied ethnographies [16,17] of integrative medicine programs at St. Michael's Hospital (SMH) in inner-city Toronto, Ontario, Canada and the CARE (Complementary and Alternative Research and Education) Program at Stollery Children's Hospital Edmonton, Alberta, Canada. An applied ethnography employs fieldwork including observation, key informant interviews and an analysis of documents to focus on a single, practical issue. [16] In this case, the focus was on understanding what makes an evolving integrative medical team successful. In each site, a series of semi-structured interviews with a range of participants (clinicians, support staff, hospital administrators) were conducted over a period of several years. In addition, the researchers observed team meetings, retreats and interaction as well as clinic operation at several stages throughout the data collection period.

At SMH, a total of 42 interviews were conducted between February 2004 and August 2006 with 18 key participants (4 administrators, 2 chiropractors, 2 physiotherapists and 10 family physicians). All participants were interviewed at least twice, with the exception of two administrators and three family physicians who were only interviewed once. All interviews included questions about perceptions of, and involvement in, the integration of chiropractic services at SMH. At CARE, 44 interviews were conducted with 24 people on four occasions: June 2004, March 2005, November 2006, and June 2007. Five participants were interviewed only once due to staff turnover. Eighteen participants were interviewed 2 – 3 times. Only 1 participant was interviewed at all four visits. During the interview, the participants were asked to reflect on their experience of working in the CARE program; specifically, what seemed to be working well, what was not working, and what had changed since the authors' previous visit.

All interviews were audio recorded and transcribed verbatim. The initial 10 interviews at each site were coded individually by both authors using basic content analysis [18-20] to identify key themes. Transcripts were then entered into the qualitative software program Nvivo 2.0 for further analysis. [21] Data from each site were collected and analyzed separately. Only when it became clear that there were many similar themes arising from the two data sets was the idea for this comparative paper formulated.

## Results

Both programs described themselves as providing integrative medicine by which they meant combining CAM and conventional medicine therapies in an evidence-based approach to providing patient care. For instance, one of the stated goals of the CARE program was to:

*...create a supportive and collaborative environment where conventional health care providers, CAM practitioners, and trainees can investigate and learn about CAM therapies and products from a rigorous evidence-based perspective*[22].

Similarly, one of the SMH program's progress reports described the project as the creation of:

*... an integrative model of care within the Department of Family and Community Medicine, with the inclusion of chiropractic services. The project team created an ongoing working group that described practitioners' scopes of practice, developed referral protocol, created reporting and communication mechanisms and supported a patient-centered, evidence based approach to care delivery*[23].

The programs both embraced Sackett et al.'s definition of evidence-based medicine being the "integration of the best research evidence with clinical expertise and patient values," (p. 1)[24].

CARE was the larger and more complex of the two programs. It consists of four main "arms": clinical, administrative, research, and education and currently includes more than 35 full and part time staff members, such as a naturopathic doctor, a traditional Chinese medicine practitioner, and a massage therapist. Situated in a large academic pediatric hospital, the research and administrative arms were the first to develop, followed closely by the education arm. The clinical arm, which is the focus of this paper, was the last to develop largely because of myriad logistical issues associated with hiring and credentialing CAM practitioners to work in an academic medical centre. The CARE integrated medicine clinic began as a referral-based, pediatric out-patient consultation service.

As it now operates, patients (and their families) referred by pediatricians are interviewed by a CARE paediatrician as well as a range of CAM providers based on patients' questions, concerns and interests. Patient cases are then discussed by the relevant CARE CAM and conventional practitioners, as well as team support staff and information specialists. Following this process, integrative consult letters summarizing what is known about the safety and efficacy of CAM therapeutic options for each individual patient are drafted. Although CARE is limited to pediatric consults, it is not limited in terms of the conditions with which patients present. At the time this is written, assessments are done by CAM providers in the CARE clinic as part of the patient's assessment; however, provision of CAM therapies is conducted off-site in practitioners' private offices.

In contrast, the SMH integrative medicine team consists of only one type of CAM provider (chiropractors) who were integrated into an existing family medicine outpatient clinic at a large inner-city academic hospital that included family physicians, nurses, physiotherapists, social workers, dieticians, occupational therapists and pharmacists. In this case, two chiropractors joined a team of two physiotherapists and 43 physicians. Currently, both the chiropractors and the physiotherapists provide on-site treatment and follow-up of adult patients referred by clinic physicians. The physiotherapists and the chiropractors have separate treatment rooms in the same office, but the physicians and other providers are located in four clinical settings set within 5 km of the hospital.

Despite the obvious contextual differences in the two programs, a striking number of similar themes emerged from the data. The five most important shared themes were: 1) the necessity of "champions" and institutional facilitators to conceive of, advocate for, and bring the programs to fruition; 2) the credibility of these champions and facilitators (and the credibility of the program being established) was key to the acceptance and growth of the program in each setting; 3) the ability to find the "right" practitioners and staff to establish the integrative teams was crucial to each program's ultimate success; 4) the importance of trust (both the trustworthiness of the developing program as well as the trust that developed between the practitioners and senior administration in the host institutions); and 5) the challenge of finding physical space to house the programs. Each theme is discussed in greater detail below.

### Champions

Many respondents highlighted the importance of key players who championed these programs. There was widespread agreement across participants that without these champions, neither the CARE nor the SMH program would exist. In the case of both programs, one or more facilitator(s) within the host institution were required in order to gain funding, space and approval for the programs. These facilitators were aided by key "champions" who became visible leaders of the programs. In both programs studied, the key champion happened to be a dynamic woman who had been working toward the development of the integrative medicine program for many years. In one setting, the champion was a physician working from "inside" the conventional medicine system. In contrast, the champion at the other site was a CAM provider. In both cases, the champion provided the passion and energy to make the integrative service happen with help of the facilitators:

It helps to have a strong advocate and a champion who keeps pushing because it keeps it on the agenda. (CARE, June 2004)

[Champion] was very, very effective in advocating with the [provincial ministry of health] and using her relationship with people at the Ministry to push that forward...I think [Champion] was extremely effective with her strategy. (Admin 1, SMH)

There are a lot of people who are interested... but I think having an interest alone is not going to be enough to get a clinic up and running. You need someone to champion it, and who has knowledge as well as the motivation. And we have that. (CARE, March 2005)

The services only became reality when these champions were able to mobilize support at a variety of different levels throughout the conventional medicine hierarchy within the institution with which they were affiliated. In both cases, the facilitators within the host institutions played essential roles in this stage.

Respondents recognized that potentially controversial programs such as these require champions who are stellar in terms of their personal and professional credibility. "Credible" champions were identified as one of the key success factors for these programs:

She [champion] is passionate about what she does, but her personal presentation I think is astute and conciliatory and not over-zealous and she is willing to listen. She has years of experience, both practically and politically, and that really comes across. I am sure that she has heard these arguments and criticisms a million times, but she deals with them graciously and politely and patiently and that kind of presentation is critical I think. It gave her a personal level of credibility.... She is very adept at getting money and convincing the powers that be, both at the hospital and ministry level, to go along with this. (Admin 3, SMH)

The adjectives used to describe the characteristics of both champions were strikingly similar. According to participants, each champion had vision, inspired both trust and confidence, and were able to mobilize many different types of people to work together. Participants also talked about the champions' energy and confidence in what they were trying to achieve, as well as their ability to include others in their plans:

...good credentials, successful, driven, very ambitious, is able to articulate a vision. (CARE, June 2004)

It was her vision. And her energy and her positivism. (Admin1, SMH)

She really gives us all a sense of equality and tries to get us involved, open minded, happy. We trust [Champion] (CARE, March 2005)

The champions, aided by the institutional facilitators, spent a great deal of time and energy laying the groundwork for the CARE and SMH programs within their respective institutions. This groundwork was instrumental in preparing clinicians and administrators at all levels of each home institution (and in the case of CARE, the regional health authority) to ensure that the integrative medicine program would be able to survive and thrive once it was introduced. Given the scarcity of resources and the range of opinions (and, in many cases, negative biases) about CAM therapies in general, it was essential that the champions and institutional facilitators were diligent in laying this groundwork prior to the initiation of the integrative medicine programs. This preliminary work made it clear that the programs were supported by senior level administration and physicians throughout the conventional medicine organization, which in turn sent the message to other clinicians throughout the institutions that these programs were safe, legitimate options for providing care for patients:

It's important with any new service. We need to pave the way, we need to market it. We need to send positive messages to the potential referral services. Physicians and family physicians tend to be somewhat conservative about where they're going to send their patients. They're protective, and they're not just going to send them anywhere. So they want to know that it's supported and endorsed and has credibility and it's going to be a safe service....And also again sending the message that senior levels of program administration supported it and had confidence in it....(Admin2, SMH)

We've also done a survey of all the divisions, like everyone who's a member of the Department of Pediatrics. That was to find out who was going to be sending patients to us. Because we wanted to be able to prepare in advance for what their clinical needs will be.... So the clinicians have an understanding that there is a need, they have a need for this knowledge, their patients have a need for this knowledge, they would love to have a place that they could send patients. It was a lot of work up front, but it's paid off. (CARE, June 2004)

### Credibility and Trust

Participants interviewed for both projects explained how acquiring credibility within the host institutions was crucial to the success of the programs. For example, the SMH project had to overcome a history of distrust and, in some cases animosity, between chiropractors and physicians. [25-28] Physicians in the SMH family medicine clinic in particular had to be convinced that this was a safe, reliable service. The reputations of both the champion and the chiropractors chosen to work in the clinic lent credibility to the existence of the program and seemed to make the physicians interviewed comfortable referring to the service:

I know their names, I know who they are, I know they're good and well trained, that makes it [referring to the service] a little easier.(Health Care Professional (HCP)6 SMH)

I think that having a reliable, credible source is very important. It's the same with anything. I don't refer to all the gastroenterologists in the city either, right. So having a reliable, quality source that's actually in the hospital, and in the hospital context, makes a huge difference.... So would I refer to every chiropractor in the city the same way? No. I don't refer to every doctor in the city the same way. (HCP9 SMH)

One of the key ways the SMH program built credibility and trust among the physicians of the host institution was by voluntarily limiting the scope of chiropractic practice to musculoskeletal complaints for which there existed a basis in scientific evidence. In addition, the chiropractors' practices were limited to referrals from physicians in the family practice unit and two other units in the hospital. Thus patients treated by the chiropractors were initially screened by physicians. These restrictions on the normal practice of chiropractic were key to creating the level of comfort necessary to initiate the chiropractic service; however, it was the competence and skill of the chiropractors working in the service that led to high levels of trust as the program evolved over time:

It's hard to know who to send your patients to. You don't always know you if can trust the person you're sending your patient to, but in this case, we don't have this problem. (HCP2, SMH)

CARE program members also identified establishing credibility for the program as being key to the program's success. One of the big challenges for CARE was that fact that they were a pediatric service and thus had to deal with the fact that there was relatively little scientific evidence about the safety or efficacy of most CAM therapies for children. Further, CARE members were conscious of the fact that their very existence might be seen as supportive of all CAM therapies. Respondents were clear in their assertion that, in order to achieve credibility, they were striving to provide a service that is based on what evidence does exist combined with the extensive clinical experience of their team. The team also had a stated focus on collecting data to generate evidence to facilitate recommendations for future patients.

I think the fact that we are here in an academic setting gives the sceptics a little bit of comfort and sends the message that probably it is a safe program.... It gives us credibility. (CARE, March 2005)

We include CAM providers, we listen to what they say, we respect them, we've created a home where they could be part of the team. That is in its very essence a form of advocacy because the system before didn't have room for that.... we do what we say we do, which is to walk that really difficult path down the middle because sometimes the evidence comes out against a particular (CAM) therapy and then we need to say so. And sometimes it comes out in favour of us and we need to say that too. (CARE, June 2007)

CARE also began by limiting the service they provided to consultation services. No CAM therapies were provided by the CAM practitioners of the CARE team; instead, they offered their expert clinical opinions to help inform what treatment options might be helpful. In addition, although the CARE team was composed of a wide variety of practitioners, it did not include a chiropractor due to the historical concerns and political controversy surrounding chiropractic treatment for children. [29] Thus, like the SMH program, the CARE team began with limited services that were later expanded as trust that the CARE practitioners could deliver evidence-based CAM increased within the host institutions increased:

I think doing this research in an academic setting...gives credibility to a field that some perceive to be soft and fluffy. And so it brings science and a language of science, questioning, using evidence, and having hypotheses and so on to the use of CAM, which has helped us expand our scope within this setting. (CARE, 2007)

### Staff: finding the "right fit"

Participants interviewed from both teams stressed the importance of finding exactly the right kind of staff. For both programs, choosing staff was not always an easy task. The "right" people had to have very strong clinical qualifications, but also be able to participate and enhance the team as a whole:

For instance, we do the 'tell us about a time you had a difference with a colleague and how you resolved it' – the question that everybody gets in every job interview. But I think it's an important question because it's a fact of life and you can tell when people answer that question if it sounds rehearsed or not. What we want is for them to come up with something really specific like 'I just didn't see eye to eye with my director on this, and we just couldn't resolve it and then I just decided that I was going to write out my piece and solve it creatively'...that's one of the things that we were looking for. This direct honesty, face to face honesty. That's when we know we have a good fit. (CARE, June 2007)

And from the first time I met them I found both (chiros) very friendly and open and not defensive and not pushy, just very collaborative. Anxious to make this project work and very positive about it, but very respectful of the fact that many people here probably were not very familiar with chiropractic. I think also the fact that they are both very experienced chiropractors helped, and on staff at the [chiropractic college], because as a person myself who knew relatively little about chiropractic in the past, I could relax about their clinical competence. I didn't have to worry about that at all, whereas somebody who was much younger and on a learning curve in their practice, not only in terms of the techniques and treatment things of chiropractic but also with dealing with difficult patients. So these individuals as choices of staff for the program was obviously a hugely important consideration. I don't know how we would be doing without them. (Admin3, SMH)

### Space

Finding space for both programs within the larger institutions was a huge issue for both initiatives. With the SMH program, the chiropractors were promised a renovated space containing two treatment rooms and a rehabilitation gym. This renovation did not get underway until more than halfway through the time allotted to the research phase of the program. While this problem was ultimately solved, space issues continued to be a factor throughout most of the pilot period and definitely slowed the development of the program:

I think one of our major limitations is going to be that we don't have enough space... and that's limited like how much patient volume we can see and our ability to practice more efficiently. Right now, I have an office on another floor and I have to run up the stairs to work on my computer and then run downstairs to see my patients. It's very limiting. (HCP2, SMH)

We call "space" the five-letter word! (Admin1, SMH)

Similarly, in the case of CARE, space was identified as one of few, and most significant, barriers to the growth of the program:

So what are our barriers right now? I'd say the first is space: physical space. I had not anticipated this...We have had such rapid expansion that finding physical space to put people: where will their desk be? Where is their computer? Do they have a phone? [These] are issues that you don't need that when you only have an idea. But suddenly when that becomes a reality, these people need stuff. So finding them a physical home has been really, really difficult. (CARE, June 2004)

## Discussion and Conclusion

Perhaps the most interesting finding to emerge from these data was the high degree of similarity between the themes associated with each program. Both programs have been successful at integrating the services of CAM and conventional medicine providers on many levels. They continue to struggle with some of the factors previously identified in the literature, including communication among team members, which in both cases is exacerbated by the large number of part-time staff, and challenges finding appropriate space where the entire team can be housed together. The preponderance of part-time practitioners is partially driven by economic incentives and is related to the fact this study tracked two integrative medicine programs from their inception. Both continue to evolve and expand, and it is likely that the number of full-time team members will increase and space issues will be more permanently resolved as the programs mature.

Our results suggest that a highly respected champion is necessary for the development of a new integrative medicine program. Both these programs were highly dependent on the efforts of champions with visions who were able to mobilize a wide range of individuals at many different levels within the health care system in order to facilitate the actualization of these integrative medicine programs. Previously, Vohra et al. identified the necessity of a "motivated champion" to initiate an integrative medical centre. [13] However, there has been little discussion of role or characteristics of such a champion prior to this study. Our findings clearly show that that the champion can be either a CAM provider or a conventional medicine provider. What is most important is that the champion has credibility within the host institution, as well as with patients and clinicians who will work together in the integrative medicine program. The champion is not necessarily the primary administrative or clinical leader of the subsequent program, but usually maintains some kind of leadership role, at least for the birth and early development of the program. Our results lead to two new research questions: 1) can an integrative medicine program evolve without a champion? And 2) what happens to integrative medicine programs when their champion leaves – can they survive?

The findings of this study highlight another necessary factor for new academic integrative medicine programs. Both evolving clinics were flexible in implementing their visions to address issues and concerns related to their unique contexts. CARE began with only a consult service and to date does not include a chiropractor among the team members despite the high use of chiropractic among children. [30] Similarly, the chiropractors in the SMH clinic began by explicitly limiting their legally-defined wide scope of practice to musculoskeletal conditions. However, as referrals and comfort levels increased over time, the SMH chiropractors have seen a wider range of referrals and increasingly have been asked to provide 'second opinion' level service when their physicians are unsure of the diagnosis (ie, because of diagnostic uncertainty, they will defer the diagnostic opinion to the chiropractors). This suggests an evolution in the physicians' level of trust and confidence towards the chiropractors. [31] In both settings, the CAM providers were being integrated into conventional medicine contexts and as such, there was a degree of conventional medicine dominance created by the existing structures as has been described previously. [9,32-34] In both clinics, the CAM providers were required to initially limit the scope of their activities in order to gain access to the integrative setting.

In both settings, it appeared that time was needed to establish the level of trust necessary to fully integrate the CAM services into the host institutions. This was facilitated by the demonstrated competency of the CAM providers and highlights how important the choice of these individuals was in each instance. Launsø identifies a range of competencies that facilitate integrative team work including: the ability to "think as a team", including the ability to cooperate, openness to working together, respectful attitude toward others and willingness to solve tasks as a group. [11] These were all highlighted in our findings as the participants described how they identified practitioners that would be a "good fit" for their teams. Although these personal characteristics and attitudes were vital to aid the growing collaborative nature of the teams, perhaps the most fundamental characteristic of all team members was their excellent clinical skills in their home discipline. These clinical skills helped build the credibility of the team as a whole and may have contributed to building trust among the integrative medicine team members.

Like all research projects, this study has limitations. Data from only two clinics are compared. However the high degree of similarity among the key themes despite the large differences in context suggests that study of other teams will not add many additional themes. Both integrative medicine clinics were followed from their inception to stable functioning; however, it is not possible to infer the long term success of either program at this time and thus, we cannot be sure the common themes we describe are in fact "success" factors.

Despite the wide contextual difference in the two clinical settings under study, there was a large overlap in the key characteristics of these integrative medicine programs which have allowed them to succeed so far. Our data suggest that champions with vision and energy supported by institutional facilitators who were able to mobilize a range of others are necessary to establish an integrative medical program. The programs arose in host institutions (St. Michael's Hospital and The Stollery Children's Hospital/University of Alberta) that were open to trying new care-delivery programs, as is evidenced by the very fact that these programs were allowed to become firmly established and thrive within their walls. The programs were ultimately successful because of the credibility of both the champions, facilitators and the staff members. Selection of excellent clinicians who were able to work well as a team facilitated the establishment of trust both within the team members as well as between the team and the host institution.

## Abbreviations

The abbreviations used in this article were: CAM: Complementary and alternative medicine; CARE: Complementary and Alternative Research and Education program; SMH: St. Michael's Hospital; and HCP: Health care practitioner.

## Competing interests

The authors declare that they have no competing interests.

## Authors' contributions

HSB conceptualized the study, participated in data collection, analysis and interpretation, helped to draft the paper and approved the final manuscript. NK participated in data collection, analysis and interpretation, helped to draft the paper and approved the final manuscript.

## Pre-publication history

The pre-publication history for this paper can be accessed here:


